# AT-0174, a novel dual IDO1/TDO2 enzyme inhibitor, synergises with temozolomide to improve survival in an orthotopic mouse model of glioblastoma

**DOI:** 10.1186/s12885-024-12631-w

**Published:** 2024-07-24

**Authors:** Michael J. Bickerdike, Imane Nafia, Alban Bessede, Cheng-Bang Chen, Medhi Wangpaichitr

**Affiliations:** 1 Antido Therapeutics (Australia) Pty Ltd, Level 7, 616 St Kilda Road, Melbourne, VIC 3004 Australia; 2 BioTarget Consulting Ltd, Auckland, New Zealand; 3 Explicyte Immuno-Oncology, Bordeaux, France; 4 College of Engineering, University of Miami, Miami, FL USA; 5 Sylvester Comprehensive Cancer Center, University of Miami, Miami, FL USA; 6 Miami VA Healthcare System, Miami, FL USA

**Keywords:** IDO1, TDO2, Glioblastoma, Tryptophan, Kynurenine, Temozolomide

## Abstract

**Background:**

Glioblastoma is an aggressive brain cancer, usually of unknown etiology, and with a very poor prognosis. Survival from diagnosis averages only 3 months if left untreated and this only increases to 12–15 months upon treatment. Treatment options are currently limited and typically comprise radiotherapy plus a course of the DNA-alkylating chemotherapeutic temozolomide. Unfortunately, the disease invariably relapses after several months of treatment with temozolomide, due to the development of resistance to the drug. Increased local tryptophan metabolism is a feature of many solid malignant tumours through increased expression of tryptophan metabolising enzymes. Glioblastomas are notable for featuring increased expression of the tryptophan catabolizing enzymes indole-2,3-dioxygenase-1 (IDO1), and especially tryptophan-2,3-dioxygenase-2 (TDO2). Increased IDO1 and TDO2 activity is known to suppress the cytotoxic T cell response to tumour cells, and this has led to the proposal that the IDO1 and TDO2 enzymes represent promising immuno-oncology targets. In addition to immune modulation, however, recent studies have also identified the activity of these enzymes is important in the development of resistance to chemotherapeutic agents.

**Methods:**

In the current study, the efficacy of a novel dual inhibitor of IDO1 and TDO2, AT-0174, was assessed in an orthotopic mouse model of glioblastoma. C57BL/6J mice were stereotaxically implanted with GL261(luc2) cells into the striatum and then administered either vehicle control, temozolomide (8 mg/kg IP; five 8-day cycles of treatment every 2 days), AT-0174 (120 mg/kg/day PO) or both temozolomide + AT-0174, all given from day 7 after implantation.

**Results:**

Temozolomide decreased tumour growth and improved median survival but increased the infiltration of CD4^+^ Tregs. AT-0174 had no significant effect on tumour growth or survival when given alone, but provided clear synergy in combination with temozolomide, further decreasing tumour growth and significantly improving survival, as well as elevating CD8^+^ T cell expression and decreasing CD4^+^ Treg infiltration.

**Conclusion:**

AT-0174 exhibited an ideal profile for adjunct treatment of glioblastomas with the first-line chemotherapeutic drug temozolomide to prevent development of CD4^+^ Treg-mediated chemoresistance.

## Introduction

Glioblastoma multiforme is the most commonly occurring tumour of the central nervous system, accounting for up to 80% of primary malignant central cancers [1] and is a deadly disease with extremely poor prognosis. Patients have a median survival of only 14–15 months from diagnosis following current best treatment approaches [2]. While surgery is often attempted it is not always possible and as glioblastomas are highly invasive, relapse following surgical excision occurs in 80% of cases close to the original tumour [3]. Surgery, if it takes place, is commonly followed by radiation treatment, which can extend survival in high-grade gliomas [4], but is not always advantageous or indicated, leaving chemotherapy as the only recourse for many patients. Temozolomide (TMZ), either used alone or in combination with radiotherapy, has become the standard-of-care chemotherapeutic drug for the treatment of glioblastomas [5] on the basis of moderately prolonging survival [6]. TMZ’s principal action is as an alkylating agent that works by methylating guanine bases in DNA at N7 and O6, leading to futile cycling of the DNA miss-match repair (MMR) system, cell cycle arrest, double-strand DNA breaks, and apoptosis [7, 8]. It should be noted that several groups have explored further actions of TMZ however, and its pharmacology appears to be quite complex. In addition to its alkylating effect, TMZ treatment is understood to activate the host immune system through the exposure and emission of damage-associated molecular patterns (DAMP) [9]. In this regard, TMZ has been shown to be pro-phagocytic to glioma cells [10] and increases the cross-priming of tumour antigen-specific CD4 + and CD8 + cells [9]. Unfortunately, despite multiple anti-tumour actions, most glioblastoma patients become resistant to TMZ. The mechanisms underlying TMZ-resistance are manifold but are commonly attributed to high expression of the repair protein O6-methylguanine-DNA methyltransferase (MGMT) [11], rapid resistance development as a consequence of drug-induced damage to the MMR pathway [12], and/or overexpression of DNA glycosylase enzymes from the base excision repair pathway, which repair TMZ-induced methylation products [13]. The mechanisms underlying TMZ-resistance and current approaches to mitigate the problem have been recently reviewed [14].

**Fig. 1:**
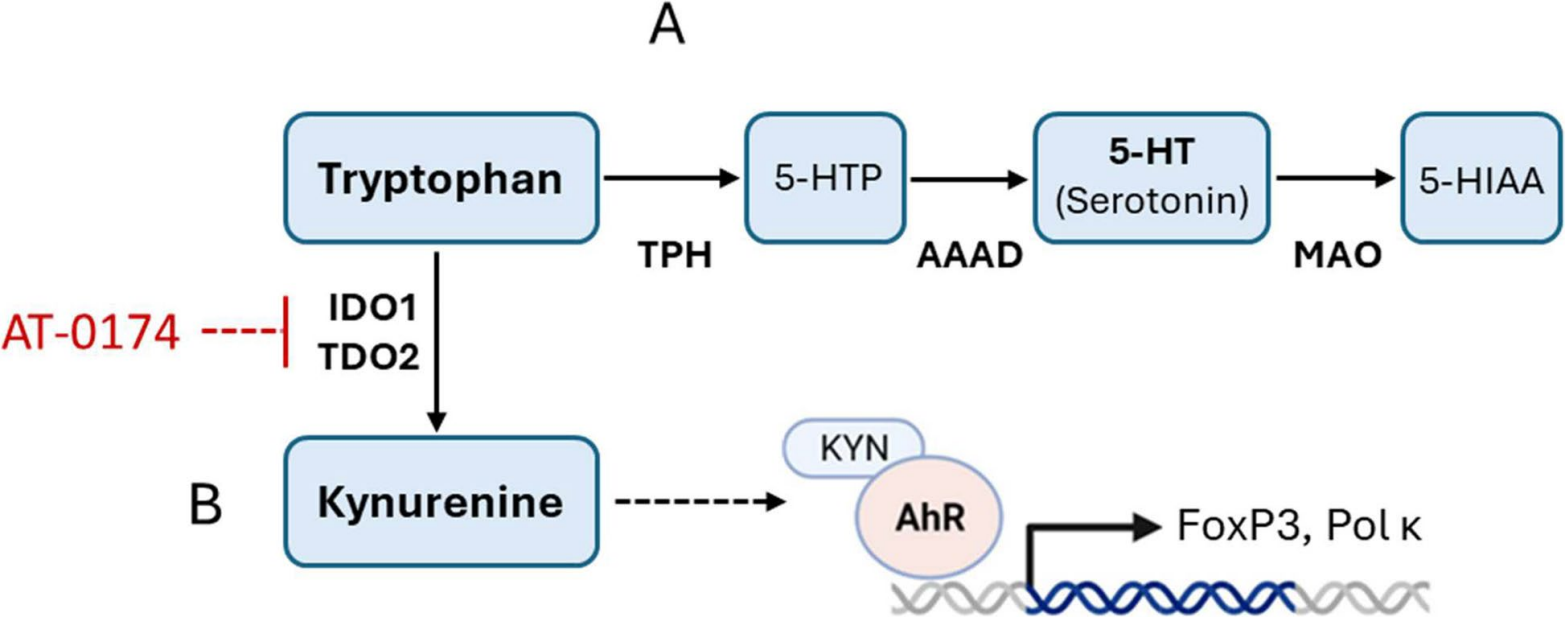
Two main paths of tryptophan (TRP) metabolism. **A **TRP to 5-HIAA conversion process is catabolized by TPH, AAAD, and MAO. **B **TRP to KYN conversion process is catabolized by two rate-limiting enzymes: TDO and IDO. TPH: Tryptophan hydroxylase; AAAD: aromatic L-amino acid decarboxylase; MAO: monoamine oxidase; IDO: indoleamine 2,3-dioxygenase; TDO: tryptophan 2,3-dioxygenase

Given the dreadful prognosis for glioblastoma patients and the usual development of resistance to first-line treatment with TMZ, new drugs are urgently required to provide improved survival and abrogate resistance to temozolomide. One promising therapeutic approach may be to target tryptophan metabolism in these tumours. Glioblastomas are notable for high expression of the tryptophan catabolising enzymes indole-2,3-dioxygenase-1 (IDO-1) and tryptophan-2,3-dioxygenase-2 (TDO2); moreover, high expression of these enzymes strongly correlates with glioma severity [15–17] and poor patient survival [15, 16, 18–20]. Enhanced expression of IDO1 and TDO2 leads to greater catabolism of tryptophan leading to raised levels of the tryptophan metabolite kynurenine in the tumour, and thereby enhancing activation of the aryl hydrocarbon receptor (AhR) for which kynurenine is an endogenous ligand (see Fig. 1). It is therefore also noteworthy that high AhR expression is also negatively linked to patient survival [20]. Tryptophan catabolism by IDO1 and TDO2 is critical within the tumour microenvironment (TME) as lowered tryptophan and raised kynurenine each confers an anti-immune response, by attenuating CD8^+^ Tcell infiltration, and promoting regulatory CD4^+^ cell differentiation [18, 21, 22]. Thus, these correlations of IDO1 and TDO2 in glioblastoma have led to a hypothesis that IDO1 or TDO2 enzyme inhibition could offer a novel therapeutic approach in the treatment of the disease [23–25].

To date, several studies have explored the efficacy of tryptophan catabolism inhibitors in rodent models of glioblastoma. The use of TMZ in these models in conjunction with selective IDO1 inhibitors such as 1-MT [26], NLG-919 [27], or PCC0208009 [28] have demonstrated the potential of IDO1 block to synergise with TMZ to improve outcome. However, while these results showed some promise, glioblastoma is especially notable for high TDO2 expression, and IDO1 inhibition in the absence of TDO2 inhibition may well have limited efficacy of these compounds in treating glioblastoma. Furthermore, there is increasing evidence to suggest that there is redundancy between the IDO1 and TDO2 tryptophan catabolising enzymes – IDO1 inhibition leads to an increase in systemic tryptophan, which in turn activates a large pool of TDO2 in the liver [29] elevating systemic kynurenine, which is likely to counteract local tumour IDO1 inhibition. Moreover, selective IDO1 inhibition can itself increase TDO2 expression in tumour cells [30]. For these reasons, it is proposed that a dual IDO1 and TDO2 inhibitor may offer an improved drug profile for glioblastoma treatment. Here, we provide data evaluating the novel dual IDO1/TDO2 inhibitor AT-0174 [30, 31], in an orthotopic model of glioblastoma.

## Materials & methods

### AT-0174 & pharmacological profile

The novel dual IDO1/TDO2 inhibitor AT-0174 was manufactured and provided for study by Antido Therapeutics (Australia) Pty Ltd. Inhibition potency at IDO1 (IC_50_ 170 nM) and TDO2 (IC_50_ 250 nM) have been described previously [30]. As a compound with pharmacophore similarity to tryptophan, the selectivity profile against other related enzymes and receptors was assessed. AT-0174 was assessed for inhibition of tryptophan hydroxylase (TH) and monoamine oxidases A and B (MAO-A, MOA-B) by spectrofluorimetric enzyme assays and for binding potency to adrenergic α_1_, α_2_ and β, dopamine D_1_ and D_2_, serotonin 5-HT_1A_, 5-HT_1B_, 5-HT_2A_, 5-HT_3_ and 5-HT_6_ receptors and serotonin and monoamine transporters, by radioligand binding assays. Radioligand binding assay conditions are summarised in the selectivity results table (see results section). Direct activation of the aryl hydrocarbon receptor (AhR) was assessed in 1A2-DRE™ cells that harboured the human AhR gene, and a luciferase reporter gene linked to the human CYP1A2 promoter. Transcriptional activation of AhR was monitored by luminescence.

### Animals

Mice were housed in an A2 animal facility, and animal studies were carried out under protocols approved by the Institutional Animal Care and Use Committee at the University of Bordeaux. The study is reported in accordance with ARRIVE guidelines. 7-week-old female C57BL/6J mice were obtained from Charles River Laboratories and acclimated for 13 days prior to use at 9 weeks of age. Ventilation was 25 volumes/hour in temperature (21–22 °C) and humidity (~ 50%) controlled conditions under 12 h/12 h light/dark. Free access to mouse pellet food and water was provided.

### Subcutaneous tumour model

#### Over-expressing IDO1 and TDO2 glioma cell lines

To assess the pharmacodynamic effect of AT-0174 in vivo, changes in plasma and tumour tryptophan and kynurenine were assessed in mice with subcutaneous syngeneic tumours derived from IDO1 and TDO2 over-expressing GL261 cell lines. This preliminary efficacy study was undertaken with the principle aim of dose exploration, with a view to select an optimal dose for a subsequent orthotopic glioblastoma model study. The murine GL261 glioma line was obtained from the National Cancer Institute (Frederick, MD, USA) cell line repository and was maintained in α- MEM (Gibco BRL, Grand Island, NY, USA) supplemented with 10% FCS and antibiotics (100 U/mL penicillin and 100 µg/mL streptomycin) in a humidified incubator, at 37 ˚C with an atmosphere of 5% carbon dioxide in air. Wild-type GL261 cells were transfected with pAd/CMV/V5-DEST with cDNA for human IDO1 (Invitrogen, Carlsbad, CA, USA) to produce lines constitutively expressing human IDO1 (GL261-hIDO1), and human TDO2 (GL261-hTDO2) (Source BioScience, Nottingham, England). Stable transfectants were maintained in αMEM with 10% FCS and selection antibiotics (2 µM puromycin). Male C57BL/6J mice were implanted subcutaneously with either GL261-hIDO1 or GL261-hTDO2 cells (1 × 10^6^ cells per site) into the left flank (under ketamine/xylazine anaesthesia) to produce syngeneic subcutaneous glioma cell tumours that overexpressed IDO1 or TDO2 enzyme, respectively.

### AT-0174 on tryptophan and kynurenine in vivo

12 days following inoculation, mice were orally dosed with either vehicle control or AT-0174 (60, 120 or 240 mg/kg gavage in 5% DMSO) and then euthanized 2 h after oral gavage. Tumour tissue was dissected and prepared for the extraction and analysis of tryptophan and kynurenine content to provide a pharmacodynamic measure of IDO1 and TDO2 enzyme inhibition. In brief, 100 µL of each plasma sample was mixed with 100 µL potassium phosphate buffer (0.05 M, pH 6) containing 100 µM 3-Nitro-L-Tyrosine (internal standard; Sigma-Aldred, St Louis, MO, USA). 25 µL of 2 M trichloroacetic acid (Merck) was added to precipitate protein, and samples were then vortexed and centrifuged at 12,000 g for 6 min at room temperature. Standard calibration curves made from serial dilution of tryptophan (Sigma) and L-kynurenine (Sigma) were prepared with the same treatment as the samples. All supernatants collected after centrifugation were transferred into micro sampling vials and analysed for kynurenine and tryptophan by HPLC (HP Agilent 1200, Agilent Technologies, Walbronn, Germany), using the method of Widmer and colleagues [32]. Kynurenine and tryptophan concentrations in samples were calculated from the standard calibration curves and the kynurenine to tryptophan ratio (K: T) was calculated by dividing the concentration of kynurenine by the tryptophan concentration.

### Orthotopic glioblastoma model

#### Cell inoculation & dosing schedule

The murine glioma cell line GL261 was genetically modified to overexpress full-length human TDO2 or human IDO1 using a CMV promoter with a geneticin-resistant plasmid (Invitrogen). The cells were cultured in DMEM supplemented with 10% FBS, 1% Penicillin-Streptomycin, and 1 mM HEPES. Stable transfection clones were selected using G418 (geneticin; neomycin analogue) at a concentration of 200 µg/mL. The cells were validated as mycoplasma-free. Before inoculation in mice, cell viability was assessed by flow cytometry and viable cell gating. A cell suspension was prepared according to the viable cell count. On the day of inoculation, analgesia was applied by intraperitoneal injection of 0.1 mg/kg buprenorphine. Mice were anesthetized using gas anesthesia with 4% isoflurane for induction and 2% isoflurane for maintenance. The scalp was shaved and anesthetized with dermal cream and the scalp was incised to reveal Bregma. GL261(Luc2) tumour cells were resuspended in sterile PBS and stereotaxically injected via Hamilton syringe (25,000 cells injected in 1 µL) 2.02 mm left of Bregma, depth 3.04 mm (striatum). The injection site was cleaned with a betadine gauze band the skin was stitched. The anesthetized mice were placed on a warming blanket and monitored until they woke up.

**Fig. 2:**
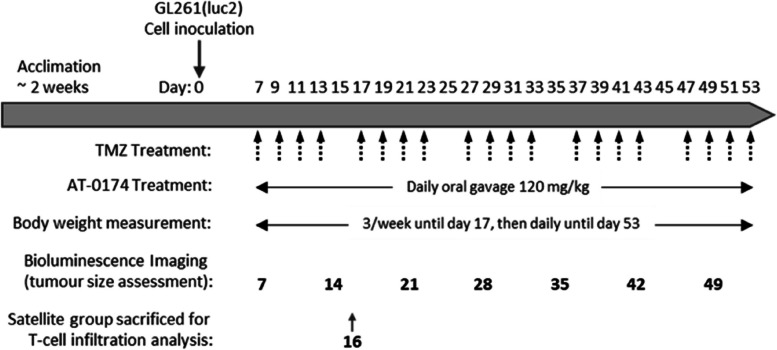
Orthotopic glioblastoma study experimental design. Following acclimation for ~ 2 weeks, female C57BL/6J mice were implanted with GL261(luc2) cells into the striatum under stereotaxic control, to provide syngeneic orthoptic glioblastomas. Drug treatments started on day 7, with temozolomide (TMZ; 8 mg/kg IP) and AT-0174 (120 mg/kg PO), given alone or in combination, on the days shown. Main study mice (*n* = 15/treatment group) were assessed for body weight, tumour growth (bioluminescence measurement) and mortality, throughout the study. A satellite group of mice (*n* = 6/treatment group) received the same drug treatment regime but were euthanized on day 16 for FACS analysis of T cell subsets to assess treatment effects on the intra-tumoural immune response

On day 6 post tumour cell inoculation, randomization and group allocation were conducted based on the first bioluminescence imaging. Animal groups, 15 mice per group in the main study and a further 6 per group for FACS analysis were treated with either vehicle control (5% DMSO), temozolomide (TMZ) (Focus Biomolecules), AT-0174 (Antido Therapeutics), or TMZ + AT-0174. The dose regimen for each treatment group is described in Table 1, while Fig. 2 shows the overall study design as a schematic.

**Table 1:** Glioblastoma study treatment groups and dose regimes

Group	Treatment	Dose^a^	Dose schedule	Route
**1**	Vehicle	-	1/day, day 7–55	PO
**2**	TMZ	8 mg/kg	Days 7,9,11,13,17,19,21,23,27, 29,31,33,37,39,41,43,47,49,51,53	IP
**3**	AT-0174	120 mg/kg	1/day, day 7–55	PO
**4**	TMZ	8 mg/kg	Days 7,9,11,13,17,19,21,23,27, 29,31,33,37,39,41,43,47,49,51,53	IP
	AT-0174	120 mg/kg	1/day, day 7–55	PO

^a^TMZ dose of 8 mg/kg was based on historical validation of this dose in the model, which has provided reliable anti-tumour effects in the authors’ hands. Alternate day dosing of TMZ with 2 day breaks after every 4th dose was also based on internal model validation and attempts to recapitulate clinical treatment regimens to some extent (i.e. frequent dosing for short periods, with breaks between)

Starting from day 6 after tumour cell inoculation, all mice were monitored in vivo until Day 55 for tumour growth (assessed by bioluminescence and measured on days 6, 14, 21, 28, 35, 42, and 49), body weight (monitored 3 times per week until day 18, and then daily from day 19–55), and survival. Mice were euthanized, according to the humane endpoints, when they showed reliable clinical signs such as respiratory distress, hunched posture, or loss of > 15% body weight.

### Grading orthotopic tumour growth

Tumour growth was measured by luciferase bioluminescence as an indicator of central GL261(luc2) cell growth. Bioluminescence readings showed a pronounced exponential increase in response to increased tumour size, with non-normal distribution, and was converted to a tumour size grade according to the following scale: BLI (ph/s/sr) 0–10,000 Grade 1; 10,001–50,000 Grade 2; 50,001-250,000 Grade 3; 250,001–1,000,000 Grade 4; 1,000,001–5,000,000 Grade 5; above 5,000,000 Grade 6. Some animals were euthanized due to tumour morbidity between days 27 and 49, and for these mice, a last-observation-carried forward (LOCF) methodology was applied to the dataset. The LOCF method represents use of the last tumour size recorded for animals on the day prior to euthanasia due to excessive tumour growth, at subsequent timepoints. This method enables more accurate assessment of drug effects on tumour size by obviating the effect of null data from dead animals at later timepoints. Such null data would falsely skew tumour growth data toward only surviving animals with small tumours.

### Immune response profiling

On day 16 post-tumour inoculation, satellite animals from each experimental group were euthanized, and tumours were collected. Tumours were processed for organ dissociation using the GentleMACS system (Miltenyi Biotec) and myelin debris removal and homogenate purification through a Percoll gradient. Tumour homogenates were then processed for immunostaining and profiling analysis by flow cytometry for the lymphoid component according to a marker panel including CD45, CD3, CD4, CD8, IFNγ, CD25, and FoxP3. For IFNγ, homogenate samples were first stimulated with a cocktail of Phorbol 12-myristate 13-acetate, Ionomycin and Brefeldin A. Briefly, to assess viability, cells were stained with LIVE/DEAD Zombie NIR (Biolegend). For simultaneous detection of the intracellular molecule, after fixed, cells were permeabilized with Foxp3 transcription factor staining buffer set according to the manufacturer’s protocol (eBioscience). All flow antibodies were obtained from BioLegend.

### Statistical analysis

One-way ANOVA was conducted for the comparison of data of different groups followed by post hoc analysis. Dunnett’s multiple comparisons post hoc analysis was used when comparing the mean of each treatment with the mean of controls in the orthotopic study. To assess the synergistic potential of AT-0174 + TMZ, this study utilized the effect size of tumour grade differences between the control and treatment groups as a metric for treatment efficacy. Effect size was calculated by dividing the mean difference of tumour grade between the Vehicle and treatment groups by the corresponding standard error (Effect size =$\:\:\frac{{\stackrel{-}{V}}_{Vehicle}-{\stackrel{-}{V}}_{treatment}}{Standard\:Error}$, where $\:\stackrel{-}{V}$ represents the mean of tumor grade). Larger effect sizes indicate a more significant treatment effect. The effect sizes were calculated for TMZ, AT-0174, and AT-0174+TMZ at Day 21, 28, 35, 42, and 49.

## Results

### AT-0174 selectivity & pharmacological profiling

AT-0174 demonstrates considerable selectivity for IDO1 (IC_50_ 170 nM) and TDO2 (IC_50_ 250 nM), with weak interactions detected at the tryptophan-related enzymes TH (tryptophan hydroxylase) and MAO-A (monoamine oxidase A) (no significant inhibition) or MAO-B (IC_50_ 6.38 µM), as shown in Table 2. No direct activation of the AhR was detected. As AT-0174 is designed to inhibit IDO1 and TDO2 by competitive binding to the tryptophan binding site of the enzymes and given the close structural similarity of tryptophan to its degradation product serotonin, AT-0174 was also screened at receptors and transporters which bind or transport monoamine transmitters. No significant binding to any serotonergic, dopaminergic, or noradrenergic sites was detected (Table 2).

**Table 2:** Selectivity profile of AT-0174

Enzyme, receptor or transporter	Expression system	[^3^H] Radioligand	Inhibitory potency	
			(µM IC_50_)	(% at 10 µM)*
IDO1 ^**§**^	hIDO1 in GL261 cells	-	0.17	100
TDO2 ^**§**^	hTDO2 in GL261 cells	-	0.25	100
Tryptophan hydroxylase	hTH enzyme assay kit	-	-	-
Monoamine oxidase A	hMAO-A in Hi5 cells	-	-	-
Monoamine oxidase B	hMAO-B in Hi5 cells	-	6.38	63
Adrenergic α_1_	Wistar rat brain	Prazosin	-	-
Adrenergic α_2_	Wistar rat cortex	Rauwolscine	-	33
Adrenergic β	Wistar rat brain	Dihydroalprenolol	-	-
Dopamine D_1_	hD_1_ in CHO cells	SCH-23,390	-	-
Dopamine D_2_	hD_2_ in CHO cells	Spiperone	-	-
5-HT_1A_	h5-HT_1A_ in CHO cells	8-OH-DPAT	-	-
5-HT_1B_	Wistar rat cortex	Cyanopindolol	-	-
5-HT_2A_	h5-HT_1A_ in CHO cells	Ketanserin	-	-
5-HT_3_	h5-HT_3_ in HEK-293 cells	GR-65,630	-	-
5-HT_6_	h5-HT_6_ in HeLa cells	LSD	-	-
Serotonin transporter	hSERT in HEK-293 cells	Paroxetine	-	18
Vesicular monoamine transporter	Human platelets	Dihydrotetra- benazine	-	-
AhR	1A2-DRE™ cells	-	-	- ^**#**^

^*^Percent shown where competitive binding inhibition > 15% reported at 10 µM

^§^IDO1/TDO2 data from Wu et al., 2023 [30]

-Interaction too weak to quantify

^#^Activation potential assessed at AhR rather than inhibition; no significant activation with AT-0174 up to 100 µM compared to positive controls

### AT-0174 on tryptophan and kynurenine in vivo

**Fig. 3:**
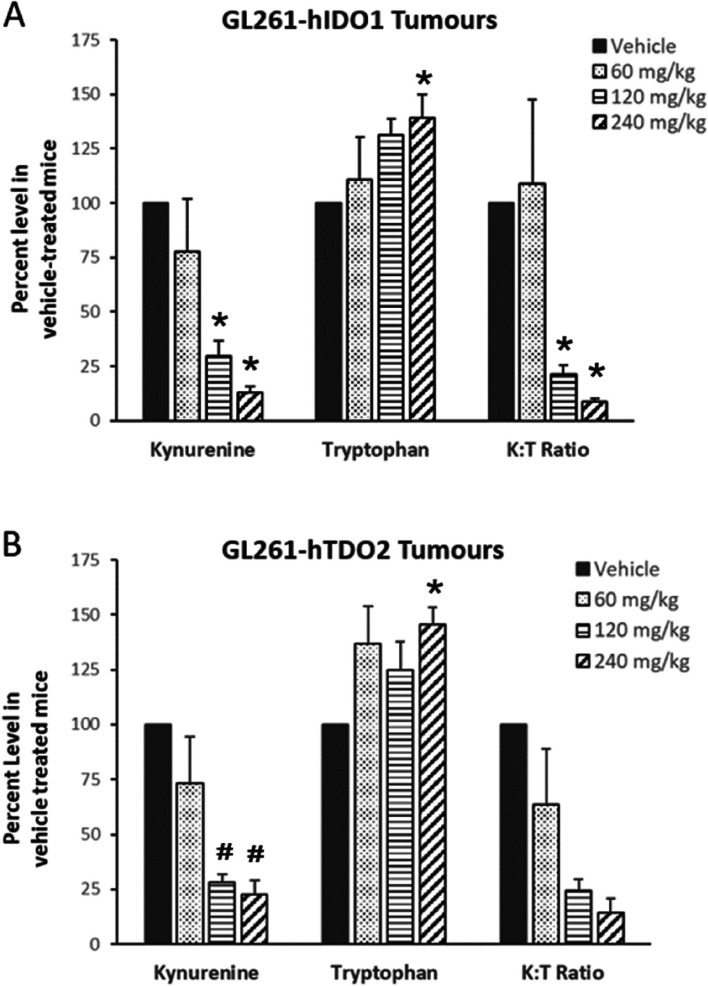
Intra-tumoural kynurenine, tryptophan and kynurenine/tryptophan (K: T) ratio in mice harbouring either (**A**) GL261-hIDO1 subcutaneous tumours, or (**B**) GL261-hTDO2 subcutaneous tumours, 2 h after treatment with oral AT-0174 (60, 120 or 240 mg/kg). * *p* < 0.01, # *p* < 0.05, according to post-hoc unpaired Student T-tests against individual control groups run for each test-article dose. *N* = 4–5 per group

Orally administered AT-0174 (60, 120 or 240 mg/kg) exerted a dose dependent increase in tryptophan and a dose-dependent decrease in kynurenine in tumour tissue dissected from subcutaneous xenograft GL261-hIDO1 tumours (Fig. 3A) and GL261-hTDO2 tumours (Fig. 3B). In each tumour condition, 120 mg/kg was the minimum efficacious oral dose, which significantly decreased kynurenine and as a consequence the kynurenine: tryptophan (K: T) ratio. The minimum oral dose required to significantly elevate tryptophan in this preliminary study was 240 mg/kg.

### Glioblastoma tumour growth

**Fig. 4:**
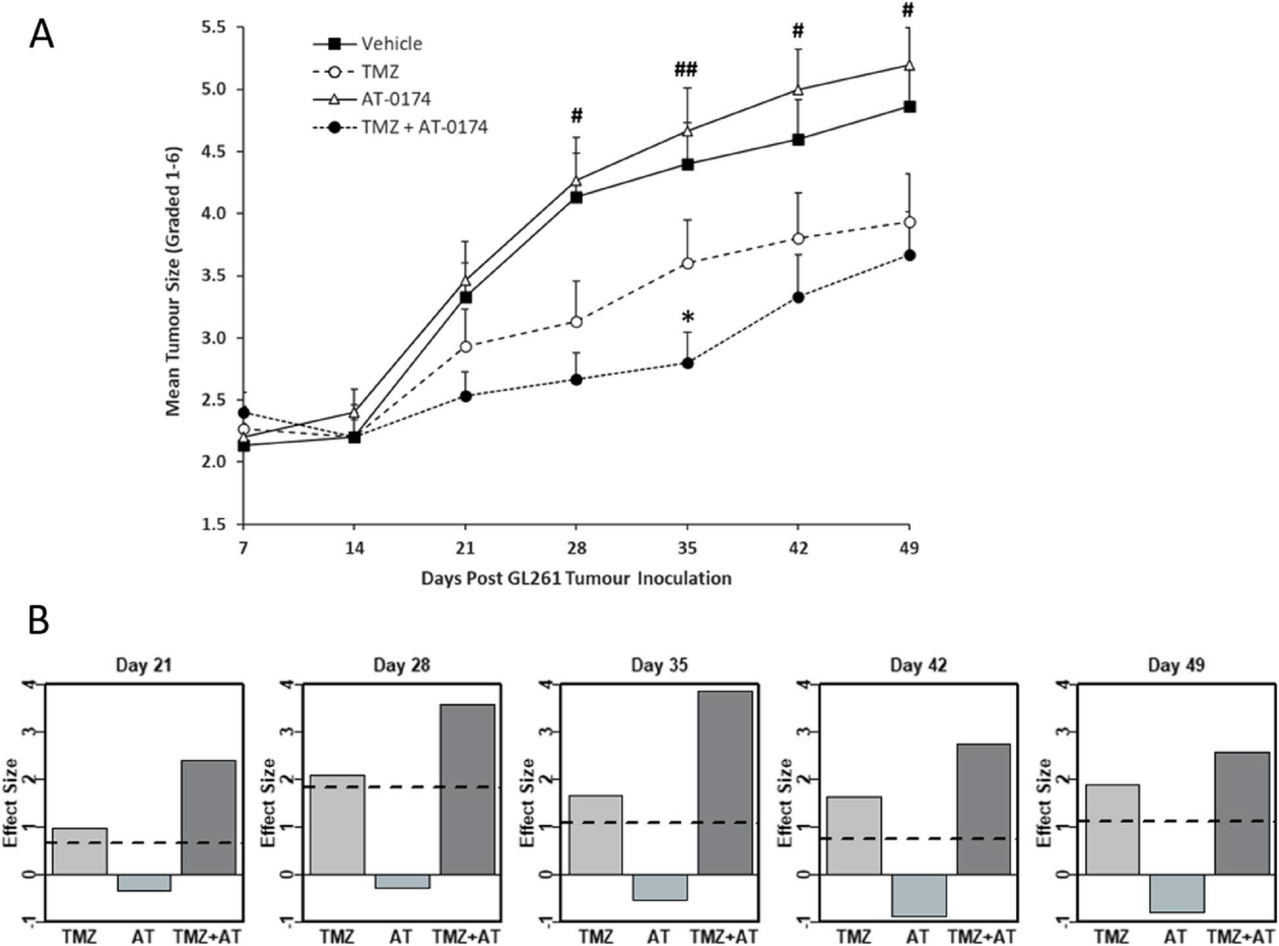
Synergy from combination treatment was observed in the drug effect on tumour growth — TMZ + AT-0174 slowed tumour growth to the greatest degree. **A** Glioblastoma tumour size was measured by luciferase bioluminescence, in female C57BL/6J mice. Mice were administered GL261(luc2) cells into the striatum under stereotaxic control and tumour growth was measured each week from day 7 after inoculation. Luciferase luminescence increased exponentially with growth and was converted into a tumour growth grade according to the following scale: BLI (ph/s/sr) 0–10,000 Grade 1; 10,001–50,000 Grade 2; 50,001-250,000 Grade 3; 250,001–1,000,000 Grade 4; 1,000,001–5,000,000 Grade 5; above 5,000,000 Grade 6. For animals that died or were euthanized due to morbidity between days 27 and 49, last-observation-carried forward was applied to the dataset. Analysis of variance (ANOVA) at each time point showed a significant effect of treatment on tumour growth on test days 28, 25, 42 and 49 (# *p* < 0.005, ## *p* < 0.001). Post hoc analysis of treatment demonstrated a significant benefit of combining TMZ with AT-0174 to decrease tumour growth (* *p* < 0.05, TMZ compared with TMZ + AT-0174). **B** The combination of AT-0174 + TMZ yielded a synergistic effect, as demonstrated by the effect size in tumor grade when compared between the control group (Vehicle) and the treatment group. A positive effect size signifies a favourable treatment outcome. The dashed line indicates a positive synergistic effect

Treatment with AT-0174 alone did not impact tumour growth. In contrast, TMZ demonstrated a reduction in tumour growth from day 21 onward when compared to the control mice treated with a vehicle (Fig. 4A). These findings illustrate the positive treatment effect of TMZ, as indicated by the positive effect size (Fig. 3B). Notably, when AT-0174 was administered in combination with TMZ, it significantly enhanced the antitumor effect. The combination of AT-0174 + TMZ exhibited a notably larger positive treatment effect than TMZ alone, highlighting a clear and positive synergistic effect within the AT-0174 + TMZ combination (Fig. 4B).

### AT-0174 on body weight in orthotopic glioblastoma mice

**Fig. 5:**
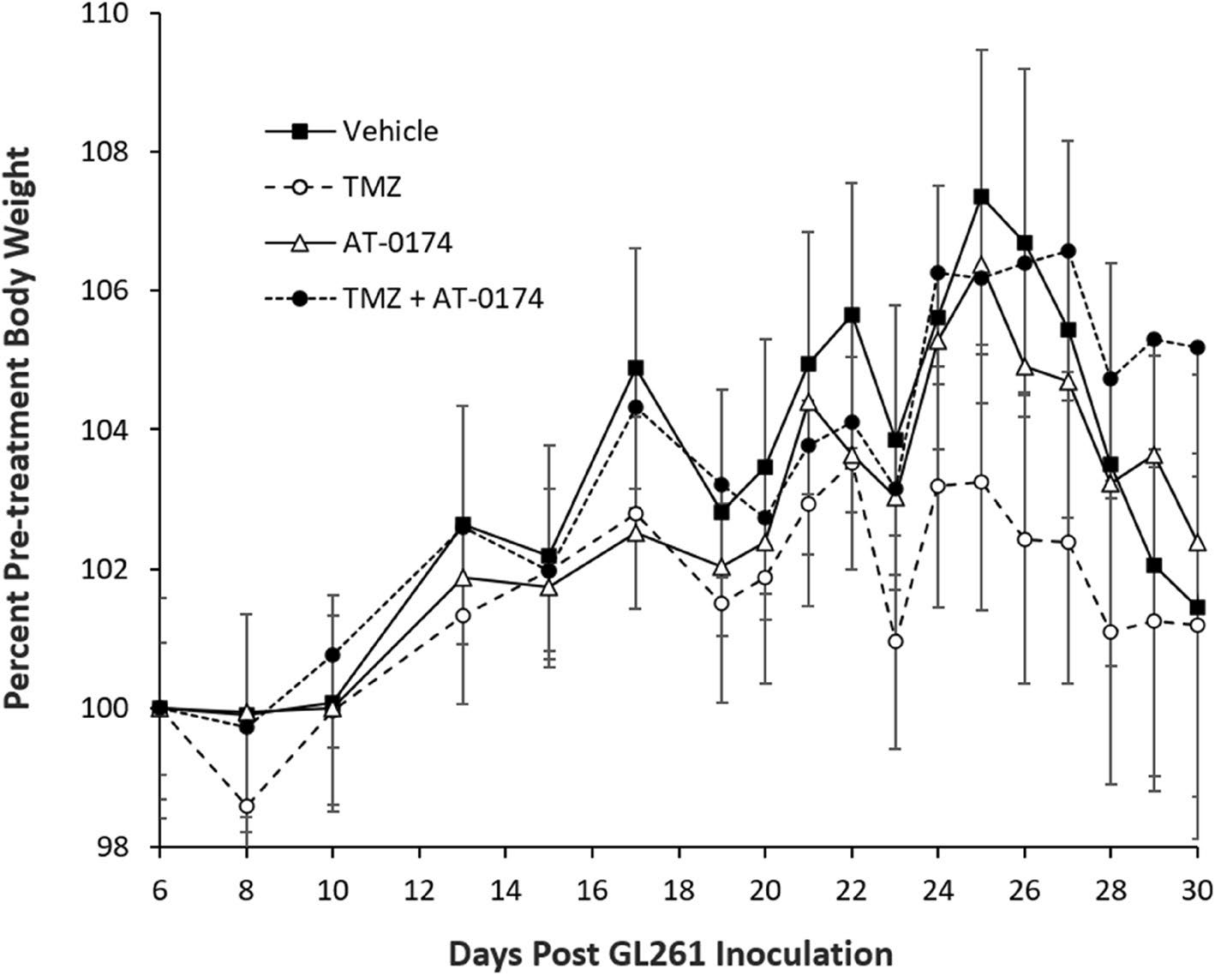
Body weight of female C57BL/6J mice orthotopic GL261(luc2) cell glioblastomas, expressed as a percentage of pre-treatment weight for each group (± SEM). Mice were treated with repeat doses of vehicle control (5% DMSO, 10 ml/kg, PO), temozolomide (TMZ, 8 mg/kg, IP), AT-0174 (120 mg/kg, PO) or a combination of TMZ and AT-0174. TMZ induced mild weight-loss in the mice relative to vehicle treated mice, which appeared to be ameliorated by combination treatment with AT-0174. Body weight measurements were taken through day-55, but escalating mortality in different treatments groups beyond day 30 led to meaningless mean body weight data and is therefore not shown

In mice with orthotopic striatal GL261(luc2) tumours, repeated treatment with TMZ appeared to induce a mild (though non-significant) drop in body weight compared to vehicle-treated animals (Fig. 5). Neither AT-0174 administered alone or in combination with TMZ had any noticeable effect on body weight. Body weight gain during the study (typically an indicator of overall health) was greatest in animals that received the combination treatment.

### Survival of mice with orthotopic glioblastomas

**Fig. 6:**
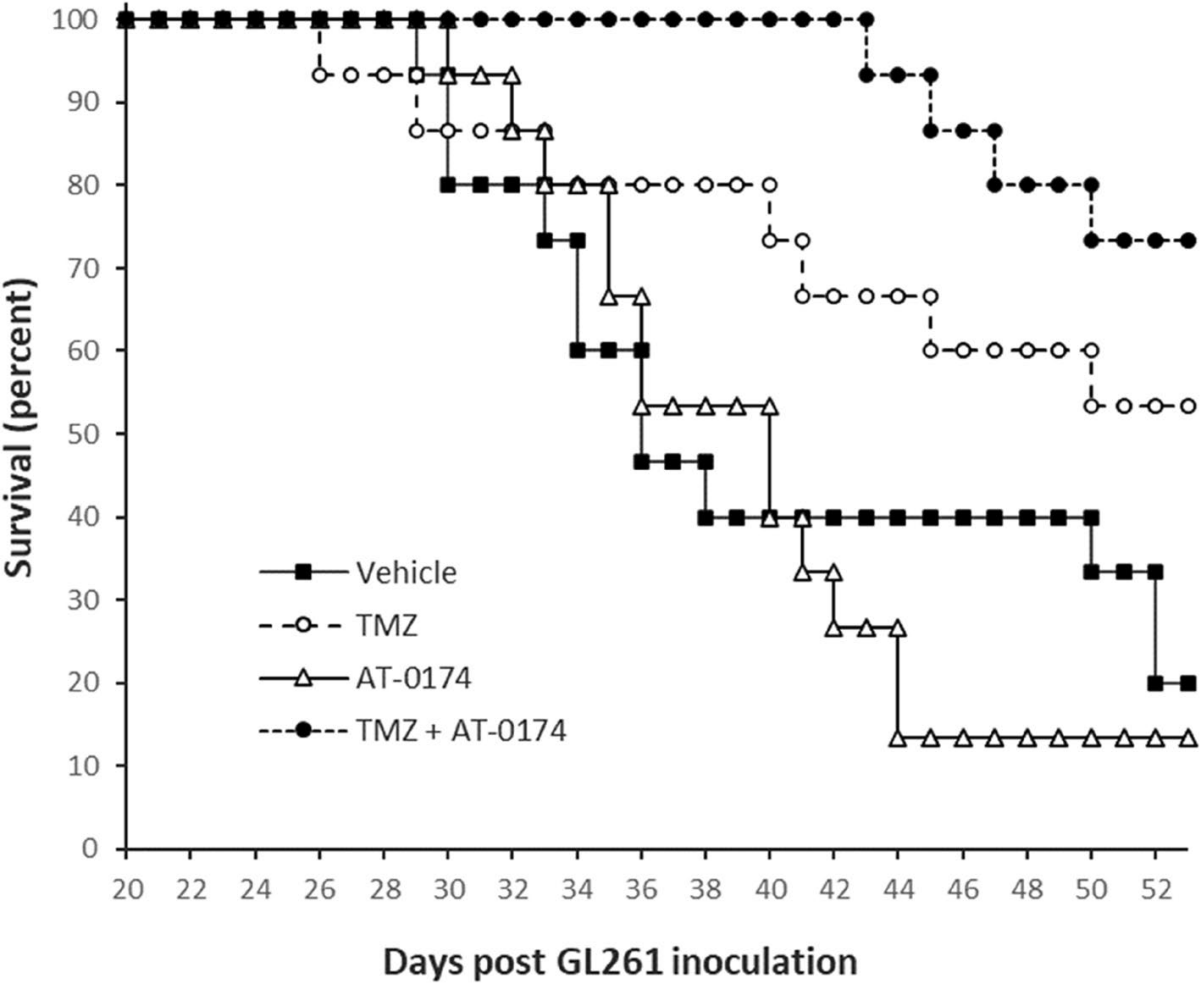
Kaplan-Meier survival curve for female C57BL/6J mice with orthotopic glioblastomas, following treatment with repeat doses of vehicle control (5% DMSO, 10 ml/kg, PO), temozolomide (TMZ, 8 mg/kg, IP), AT-0174 (120 mg/kg, PO) or a combination of TMZ and AT-0174. Statistically significant survival benefit was conferred by combination treatment with TMZ plus AT-0174 (*p* < 0.01 compared with vehicle-treated group), but not with either drug treatment when given alone. Median survival for each treatment was as follows: Vehicle = 36 days; AT-0174 = 40 days; TMZ = 54 days; AT-0174 + TMZ = undefined (73% survival at 54 days)

Vehicle-treated mice with GL261(luc2) tumours in the striatum had a median survival duration of 36 days post-inoculation, with the first mortality recorded on day 29. Daily treatment with 120 mg/kg AT-0174 by oral gavage did not significantly affect survival, with the first death on day 30, and a median survival of 40 days (Fig. 6). TMZ, by comparison, improved median survival duration to 54 days, though the first death occurred as early as day 26. Combination treatment of TMZ plus AT-0174 exerted the greatest survival benefit, with zero mortality recorded until day 44. A median survival duration could not be calculated for the combination treatment as more than 50% of the mice in the combination treatment group survived to the end of the study (74% survival on day 54). ANOVA assessment confirmed a statistically significant effect of the combination treatment on the survival of mice harbouring glioblastomas in this study (*p* < 0.01).

### Effect of treatment on immune cell infiltration

**Fig. 7:**
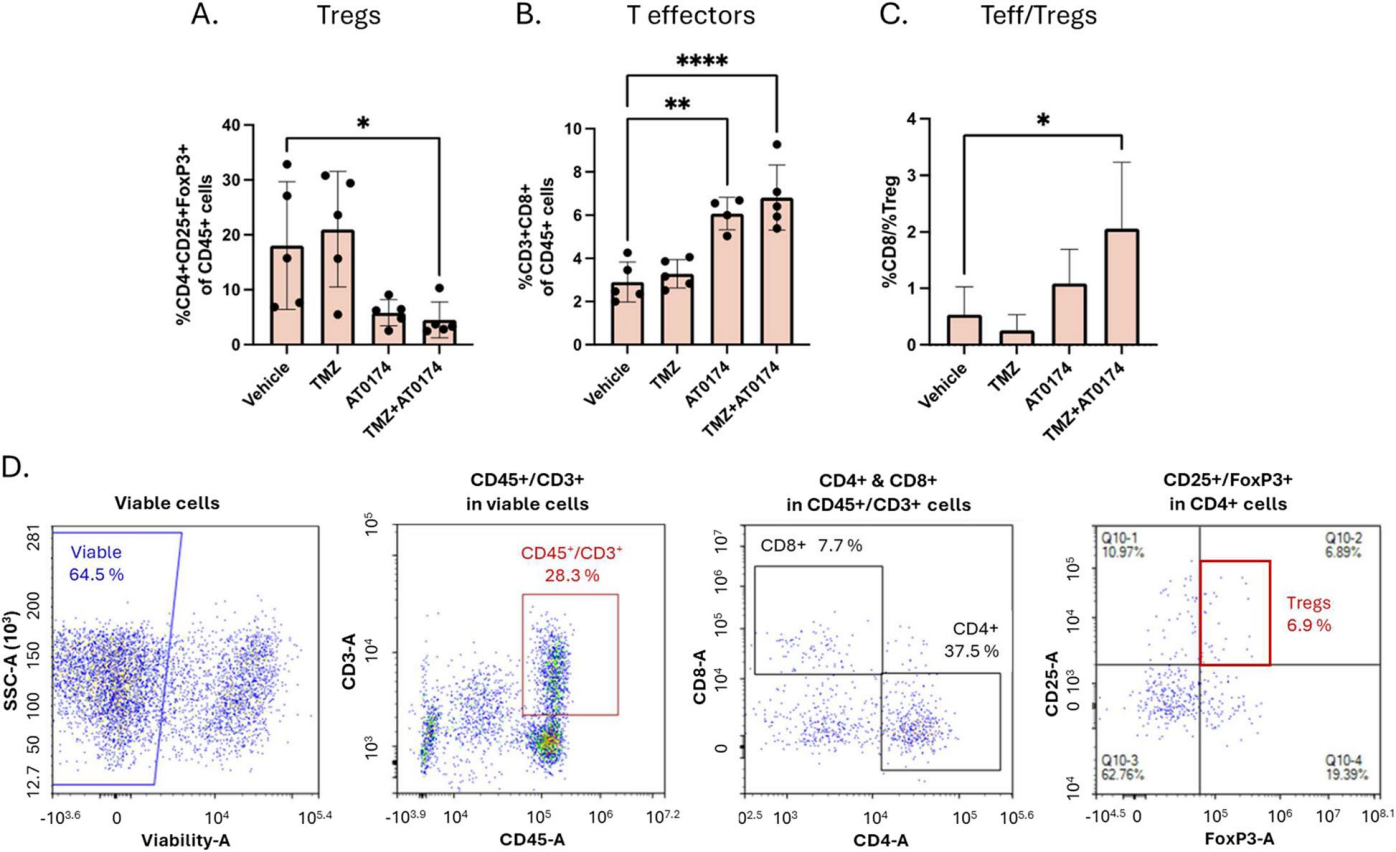
Effect of temozolomide (TMZ), AT-0174, or a combination of TMZ plus AT-0174 on the expression of T cell subsets within GLK261(luc2) glioblastomas in C57BL/6J mice (*n* = 5). **A** TMZ raised the population of regulatory T cells (CD4^+^CD25^+^FoxP3^+^ cells), whereas AT-0174 or a combination of TMZ + AT-0174 significantly decreased the Treg population (effect of treatment by ANOVA, *p* = 0.02). **B** Treatments increased the population of tumour infiltrating CD8^+^ T cells, with combination-treated mice showing the most increase (effect of treatment by ANOVA, *p* = 0.001). **C** The ratio of CD8 + to Treg cells. The pro-immune CD8^+^ to Treg ratio increases with AT-0174 administration alone, but demonstrates an even greater increase following administration of the combination TMZ + AT-0174 (effect of treatment by ANOVA, *p* = 0.01). **D** Gating strategy used to identify CD8^+^ T effector cells and Treg; viable cells were gated based on SSC-A versus Fixable Viability Dye; cells were gated on CD45^+^/CD3^+^ and further divided into CD8^+^ and CD4^+^ fractions; CD25^+^ and FoxP3^+^ cells were selected from the CD4^+^ fraction. (*p < 0.05, ***p < 0.001)

The analysis of tumour-infiltrating lymphocytes (TIL) in GL261 orthotopic mice was conducted using immune marker FACS analysis after 16 days of treatment. Interestingly, Tregs (CD4^+^CD25^+^FoxP3^+^ cells) were higher in TMZ-treated mice when compared to vehicle control. Nevertheless, AT-0174 is able to suppress Tregs when given alone or in combination with TMZ (Fig. 7A). TMZ treatment resulted in a non-significant increase in the population of cytotoxic CD8^+^ T cells when compared to control mouse tumours. However, AT-0174 treatment alone led to an increase in CD8^+^ T cells, and this increase was significantly enhanced when AT-0714 was combined with TMZ treatment (Fig. 7B). The significant increase in the CD8^+^/Treg ratio observed in the combination treatment aligns with the marked tumour inhibition demonstrated in Fig. 5. We observed the most pronounced pro-immune effect with a 4-fold increase in the CD8^+^/Treg ratio when comparing the combination treatment to the vehicle-treated mice (Fig. 7C).

## Discussion

The importance of over-expression of the tryptophan catabolising enzymes IDO1 and TDO2 in solid cancers has been known for some time [15, 33–35], with particular focus placed, until recently, on IDO1 as a potential target for cancer treatment. Increased IDO1 expression in most solid cancers, coupled with the knowledge that decreased tryptophan and increased kynurenine resulting from IDO1 over-activity suppresses the immune response to cancer cells led to a concerted effort to discover and develop selective IDO1 inhibitors as anticancer drugs. These drug candidates included the selective IDO1 inhibitors epacadostat, navoximod (GDC-0919, NLG-919), BMS-986,205, and PF-06840003. Unfortunately, the failure of these experimental, selective, IDO1 inhibitors to reach positive outcomes in clinical trials led to a decreased interest in selective IDO1 inhibitors as anticancer agents. However, the importance of TDO2-mediated tryptophan metabolism in cancer biology has been known for some time [33, 36], with TDO2-mediated production of kynurenine reported to promote tumour cell survival through activation of the AhR [19]. Additionally, there is considerable mechanistic evidence that inhibition of only IDO1 and not TDO2 activity is likely to limit efficacy, due to systemic tryptophan homeostasis (discussed below). On that basis, it was hypothesised that dual IDO1/TDO2 inhibition may offer improved efficacy in many cancers, especially in those that commonly over-express TDO2. Glioblastoma is such a cancer, with both IDO1 and TDO2 expression elevated in most patients [15, 19, 37, 38], and a strong correlation between TDO expression and poor patient survival [15, 17].

Several previous studies have investigated the effect of selective IDO1 inhibitors in combination with TMZ in mouse models of glioblastoma. In such models, glioma cell lines are typically implanted stereotaxically into the brains of mice to create an orthotopic glioma tumour and are then treated with vehicle or TMZ alone in conjunction with a test article IDO1 inhibitor. Hanihara and colleagues [26] reported a slight increase in the survival duration of mice given orthotopic intracranial GL261 cell tumours when they administered TMZ in combination with the weak IDO1 inhibitor 1-methyltryptophan (1-MT), compared to TMZ alone. In this study, 1-MT was ineffective when given alone in both the orthoptic GL261 model and in a U87 cell xenograft model. Similar results were obtained with the selective IDO1 inhibitor NLG-919 [27], which slightly improved survival when given in conjunction with TMZ. Likewise, PCC0208009, a potent IDO1 inhibitor, significantly synergised with TMZ to reduce subcutaneous GL2612 tumour growth in mice and improved survival in a C6 orthotopic rat model of glioma [28]. The activity of PCC0208009 at the TDO2 enzyme is not reported.

In the current study, the novel and potent dual IDO1/TDO2 enzyme inhibitor AT-0174, was assessed for its capacity to synergise with TMZ in an orthotopic mouse model of glioblastoma. In a preliminary dose-ranging study, oral AT-0174 was determined to elevate tumour tryptophan and significantly attenuate tumour kynurenine in glioma cell-based xenograft malignancies that over-expressed IDO1 and TDO2. These results confirmed the capacity of AT-0174 to significantly block IDO1/TDO2 enzymes to abrogate tryptophan turnover in a functionally relevant manner. These data agree with similar results observed following oral AT-0174 on serum tryptophan and kynurenine in a mouse model of lung cancer [30].

In the orthotopic GL261 glioblastoma study, AT-0174 had no effect on tumour growth or survival of mice when given alone. Interestingly, Ladomersky and colleagues [38] similarly report no effect of the selective IDO1 enzyme inhibitor BGB-5777 when given as monotherapy. The model recapitulates an aggressive and typically intractable cancer, so it is perhaps not surprising that IDO1/TDO2 inhibition alone does not confer a positive disease outcome. It should also be noted that the incorporation of a luc2 motif in the GL261 cells has been reported to impart some immune activation in the murine GL262 glioblastoma model [39]. It is therefore possible that the effect of AT-0174 alone may be confounded to some degree by the luc2 system. However, the effect of luciferase proteins on immune response is not universally reported and has not been seen by the authors in other luc2-incorporated tumour models (i.e. in a 4T1 model; data not shown). Moreover, the luc2 motif was present in all treatment groups and is therefore a well-controlled aspect of this commonly used expression system model.

In contrast to AT-0174 alone, the combination of AT-0174 with TMZ provided synergy to significantly enhance the cytotoxic effects of TMZ alone, leading to a marked suppression of tumour growth and improved survival. Several mechanisms may contribute to the observed benefits of this combination treatment. T-cell analysis of tumours from satellite groups of mice on day 16 revealed that AT-0174 reduced Treg numbers while increasing the activation of CD8 + T cells, resulting in a substantial shift away from a predominantly regulatory T-cell (Treg) population seen in vehicle-treated and TMZ-treated animals. Importantly, the CD8+/Treg ratio increased in the AT-0174 treatment group, and this increase was significantly enhanced in the combination treatment. These pro-immune alterations within the tumour microenvironment (TME) align with our recent findings regarding AT-0174 in a mouse model of cisplatin-resistant lung cancer. In that study, the dual inhibitor similarly led to a substantial decrease in Treg and myeloid-derived suppressor cells (MDSC) populations while concurrently increasing the immune effector NK and CD8^+^ T cells [30].

The effect of AT-0174 on the TME seen here meets literature expectations for an IDO1/TDO2 blocker [40], which predicts enhanced CD8^+^ activation via tryptophan-mediated suppression of GCN2 and mTOR/PKC-Θ activation [41, 42] and to suppress Treg differentiation following a drop in kynurenine-mediated activation of AhR, which in turn attenuates AhR-mediated CD25^+^/FoxP3^+^ differentiation [43, 44]. The role of AhR in immune suppression is complex, as it appears that AhR expression is exacerbated not only by kynurenine activation and GCN2 but also by tryptophan deprivation and increased sensitization to kynurenine [45]. These recent data suggest a two-pronged mechanism by which elevated IDO1/TDO2 drives immune suppression.

Importantly, depletion of Tregs has previously been directly linked to survival prolongation in mice with experimental gliomas [46], while CD4^+^/CD25^+^/FoxP3^+^ Treg infiltration positively correlates with clinical glioma tumour grade [47]. It is also worthwhile noting that promoting CD8^+^ Tcell expression is believed to be mediated by raised tryptophan, while the suppression of Tregs is believed to be mediated by attenuated kynurenine. In mouse knockout studies, it has been shown that IDO KO fails to increase plasma tryptophan, while TDO KO exerts significant effects on both tryptophan and kynurenine [48]. Dual IDO1/TDO2 inhibition is perhaps important to increase CD8^+^ Tcells and downregulate Tregs, as observed. In this manner, AT-0174 appears to markedly improve the immune profile to favour the treatment of glioblastoma.

It is interesting to observe that pro-immune cellular effects in the TME were observed in AT-0174 treatment mice, though this mono-therapy treatment did not improve tumour growth or survival outcome. A possible explanation is that an enhanced immune response may only exert benefit in animals where cancer cells are also undergoing apoptosis resulting from TMZ treatment. Furthermore, it may be the case that the pro-immune effects on T cells only represent part of the mechanism underlying AT-0174’s synergy with TMZ. This idea receives some support from the literature. TMZ is a first-line chemotherapeutic agent used in the majority of glioblastoma clinical cases, providing short-term efficacy (extending survival by 9 months or so [5, 6] through its action as an alkylating agent, methylating guanine bases in DNA at N7 and O6. This methylation ultimately leads to futile cycling of the DNA miss-match repair (MMR) system, producing cell cycle arrest, double-strand DNA breaks, and apoptosis of tumour cells [7, 8]. Drug resistance almost always rapidly develops to TMZ, however, either from high expression of the repair protein O6-methylguanine-DNA methyltransferase (MGMT) [11] or resulting from TMZ-induced damage to the MMR pathway, preventing the futile cycling that leads to tumour cell apoptosis [12, 49].

## Conclusion

To support the notion of DNA damage tolerant efficacy, we previously showed that AT-0174 is highly efficacious in vivo in mouse models of cisplatin-resistant lung cancer [30]. Here, we extend these findings by assessing the dual inhibitor in an orthotopic mouse model of glioblastoma, revealing striking synergy with TMZ, the first-line chemotherapeutic agent used in the clinic. A shift to an immune effector CD8^+^ T cell, while reducing Treg populations likely underlies some of the efficacious benefits of AT-0174. This study supports the use of dual IDO1/TDO2 inhibitors such as AT-0174 as effective treatments to be used in combination with temozolomide to improve prognosis in this intractable disease.

### Future directions and limitations

It is known that glioblastomas are characterised by elevated expression of translesion DNA polymerases, including Pol κ, Pol ι, and Pol η [50]. These polymerases are highly inaccurate and lack proofreading ability, leading to spontaneous mutations, driving genomic instability and tumorigenesis [51]. Notably, Pol κ also mitigates double-strand breaks and reduces sensitivity to DNA-damaging drugs such as TMZ [52]. The reduction in double-strand breaks likely results from Pol κ-induced hypermutation, damaging the MMR system. Wang and colleagues [50] have reported that Pol κ and Pol ι expression are highly correlated to disease stage severity in glioblastoma and are negatively correlated with survival. Peng and colleagues [53] have extended these findings, demonstrating that over-expression of Pol κ in A172 glioma cells leads to a significantly worse outcome in response to TMZ treatment in a rodent glioblastoma tumour model. In contrast, Pol κ depletion significantly extended survival in an orthotopic model of glioma [53]. Importantly, it has been shown that Pol κ is not only associated with glioblastoma outcome but is induced directly by TMZ [53]. Thus, TMZ appears to upregulate Pol κ, inducing resistance to the drug.

**Fig. 8:**
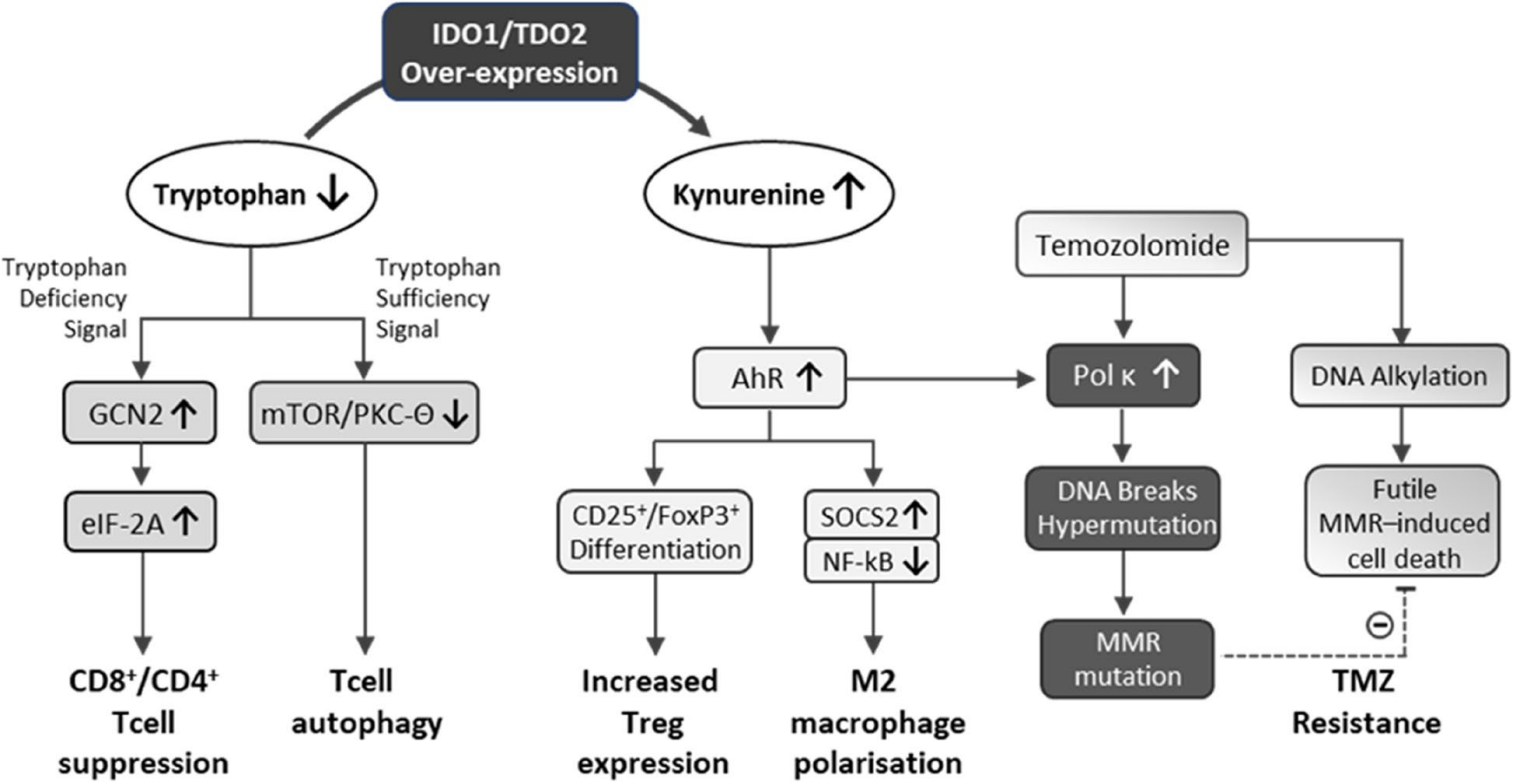
A simplified schematic overview of some of the mechanisms instigated by IDO1/TDO2 over-expression in glioblastoma, that lead to T cell suppression, Treg upregulation, and resistance to TMZ. IDO1 and/or TDO2 overactivity in the tumour microenvironment decreases tryptophan and increases kynurenine. Lowered tryptophan levels activate the tryptophan deficiency signal enzyme GCN2, which suppresses CD8^+^/CD4^+^ T cells. In addition, the mTOR/PKC-Θ mediated tryptophan-sufficiency signal pathway is down-regulated, leading to T cell autophagy and anergy. Concomitant with these changes resulting from low tryptophan, high kynurenine activates the AhR to increase CD25^+^/FoxP3^+^ differentiation, potentiating the expression of Tregs. AhR activation also leads to the polarisation of macrophages from the cytophagic M1 phenotype to the immune-suppressing M2 phenotype, via a SOCS2/NFκB pathway. Finally, in glioblastoma, kynurenine-induced AhR activation also upregulates the DNA translesion enzyme Pol κ, exacerbating induction of this enzyme by TMZ itself, to accelerate TMZ-resistance through hypermutation, attenuating futile MMR cycling and tumour cell apoptosis. It can be seen that IDO1/TDO2 over-expression therefore suppresses the immune response and encourages tumour cell growth through numerous mechanisms. A dual IDO1/TDO2 inhibitor would be expected to prevent all these mechanisms, improving the immune response to tumour cells and slowing resistance to TMZ.

Of relevance to the current study, it has been reported [53] that kynurenine signalling through AhR significantly increases the expression of Pol κ. Kynurenine responsible for activating AhR in glioblastoma cells to increase Pol κ has been shown to result from TDO2 activity, as it is prevented by the selective TDO2 inhibitor 680C91 [54]. Overexpression of TDO2 in glioma leads to raised kynurenine providing a key mechanism by which Pol κ is also raised in glioblastoma cells. Overexpression of TDO2 in addition to the Pol κ-inducing effects of TMZ itself leads to a ‘perfect storm’ wherein drug resistance is readily conferred leading to an extremely poor prognosis. In this context, it is clear how the combination of a dual IDO1/TDO2 inhibitor with TMZ treatment might be expected to result in the synergy observed in the current study. Block of IDO1 and TDO2 may therefore have a two-fold beneficial effect on tumour progression: (i) to down-regulate Treg expression, while promoting CD8^+^ cell activation to engage an immune response to the tumour, and (ii) to block kynurenine-AhR-induced Pol κ, attenuating TMZ-induced drug resistance and improving prognosis in response to chemotherapy (Fig. 8). Further work is therefore planned to directly assess the role of Pol κ inhibition by AT-0174 in its synergy with TMZ.

## Data Availability

No datasets were generated or analysed during the current study.

## Abbreviations

AhR: Aryl hydrocarbon receptor
IDO1: Indole-2,3-dioxygenase-1
MAO-A: Monoamine oxidase A
MAO-B: Monoamine oxidase B
MMR: DNA miss-match repair
MGMT: O6-methylguanine-DNA methyltransferase
TDO2: Tryptophan-2,3-dioxygenase-2
TH: Tryptophan hydroxylase
TIL: Tumour-infiltrating lymphocytes
TME: Tumour microenvironment
TMZ: Temozolomide
Treg: Regulatory T-cell
